# Evolution of Bovine Herpesvirus-1 infection prevalence and infection dynamics in Irish dairy herds following an IBR hyperimmunisation vaccination protocol

**DOI:** 10.1186/s13620-025-00328-w

**Published:** 2025-12-23

**Authors:** Ryan Duffy, Tadhg Gavin, Alistair Couper, John Quille, David McLaughlin, Emma Hanly, Christopher McGrath, Declan Gordon, Patrick Clerkin, Marina Solé Berga, Héctor Santo Tomás

**Affiliations:** 1 Precision Microbes, Curragh West Naul, Co., Dublin, K32 EP97 Ireland; 2 XLVets Ireland, Newport, Ireland; 3 Hipra UK & Ireland, Nottingham, UK; 4 HIPRA, Amer, Spain

**Keywords:** Bovine Herpesvirus-1, Dairy herds, Herd seroprevalence, Hyperimmunisation protocol, Infectious bovine rhinotracheitis, Ireland, National eradication, Vaccination, Within-herd seroprevalence

## Abstract

**Background:**

Bovine Herpesvirus-1 (BoHV-1) is endemic in the Irish cattle population, but there is no current formal eradication programme for this disease in the Republic of Ireland. This observational, prospective field study is aimed to assess the evolution of the BoHV-1 seroprevalence on fifteen Irish dairy farms (85–250 cows) between 2020 and 2023 following the implementation of a hyperimmunisation IBR vaccination programme. Eligible farms had positive bulk tank results and estimated within-herd seroprevalence ≥ 25%. Animals from three months of age received a live, monovalent, double-deleted (gE-/tk-) marker vaccine (six-monthly boosters). Annually, biosecurity was evaluated using a 44-item survey. Each year, approximately 20 randomly selected animals per farm (4 calves, 4 heifers, 12 cows) were tested by gE-ELISA; in addition, animals with a negative result were retested in subsequent years (*N* = 818 animals). Seroprevalence was analysed using a logistic regression model with year and the epidemiological unit (calf, heifer or cow) as response variables, and farm as a random effect. A Pearson correlation between within-herd seroprevalences and biosecurity scores was also performed.

**Results:**

The overall estimated animal level seroprevalence decreased from 55.7% to 37.2% after one year and was maintained at 37.5% after two years. Among the eleven herds that followed the hyperimmunisation IBR vaccination protocol, the estimated animal level seroprevalence was reduced from 57.3% to 33.6%, and the estimated within-herd seroprevalence from 54.5% to 22.2% within the study period. Reductions were most evident in calves (72.0%) and heifers (76.6%), compared with adult cows (33.1%). A significant negative correlation was observed between within-herd seroprevalences and biocontainment scores (*R* = -0.51, *p* < 0.001). Most retested animals maintained a seronegative status across the study period. However, data on culling and replacement rates were not available on the studied farms.

**Conclusion:**

The control and eradication of BoHV-1 from the Republic of Ireland present many challenges to the Irish dairy industry. The results suggest that the maintenance of a hyperimmunisation protocol with an IBR gE-/tk- marker vaccine contribute to effectively and efficiently reducing BoHV-1 seroprevalence within the Irish dairy sector at farm level. Further large-scale controlled studies are required to confirm its role in national eradication strategies.

**Supplementary Information:**

The online version contains supplementary material available at 10.1186/s13620-025-00328-w.

## Background

Bovine Herpesvirus-1 (BoHV-1), a significant veterinary pathogen [1], has become endemic in much of the global cattle population since its identification in the 1950s [2], except for a few European regions which have successfully implemented programmes to eradicate the virus [3]. Specifically, in countries with medium or high prevalence, such as France, Hungary, some regions of Italy, Slovakia, Belgium, the Netherlands, or more recently Spain, control programs have implemented the use of marker vaccines followed by the removal of seropositive animals [3, 4]. Currently, Animal Health Ireland (AHI) is assessing the feasibility of a national eradication programme in the Republic of Ireland (RoI) [3, 5, 6], aiming to advance disease control and eradication across the island [7]. This initiative is driven by the substantial negative impacts of BoHV-1 on animal health, animal productivity, and international trade [3], with an estimated annual loss of €88 million for the national dairy industry [8]. Thus, in anticipation of a potential eradication programme, only marker vaccines are licensed in the RoI against IBR since the ban on non-marker vaccines in 2004 [3, 9]. Widespread infection is evident in both the Irish dairy and beef sectors [10–16], with recent estimates showing an 80% dairy herd seroprevalence [16].

BoHV-1 eradication programmes must consider viral latency and reactivation, key features of this alphaherpesvirus which challenge disease control and eradication efforts from a defined population. Infected animals experience a persistent lifelong infection, despite the animal mounting an active immune response. Clinically ‘*recovered*’ animals fail to eliminate the virus, which remains ‘*latent*’ in the neural system, primarily in the trigeminal ganglion [1]. These animals act as reservoirs, excreting the virus intermittently during periods of stress [17], such as around calving, transportation, treatment with high doses of immunosuppressive medications [18, 19], commingling of animals from multiple sources, nutritional stressors, lameness, and many other disease processes [20]. This process is known as ‘*viral reactivation*’ and is responsible for the survival of infection within a herd and the source of subsequent viral circulation [21, 22]. A primarily infected animal can infect up to seven of its herd mates (i.e., a basic reproduction number R_0_ ≥ 7) [23], and airborne transmission is possible at 4 m, highlighting its highly contagious nature; however, a distance over 4.4 m is probably sufficient to reduce R_0_ to < 1 [22]. These figures present a significant challenge in the battle against BoHV-1 to a national dairy industry which has experienced significant expansion in response to the abolition of the European milk quotas in April 2015. Promoted under Food Harvest 2020 [24], Irish dairy cow numbers increased by 39.6% between 2013 [25] and 2023 [26] according to the Central Statistics Office (CSO), with Teagasc’s National Farm Survey reporting a 41% increase in herd size between 2014 and 2022 [27]. Multiple studies have identified increasing herd size as a risk factor for an increasing BoHV-1 seroprevalence [14, 28, 29], further complicating national efforts to control and eradicate BoHV-1.

BoHV-1 eradication efforts typically combine biosecurity improvements, routine herd vaccination, diagnostic surveillance, and strategic culling of seropositive animals [3]. In response to the high BoHV-1 seroprevalence in RoI, the AHI Annual Report 2021 suggested restricting transportation to seronegative animals only and promoted complete herd vaccination protocols using glycoprotein E (gE)-deleted marker vaccines for all herds with a BoHV-1 seroprevalence greater than 15% (deemed an indicator of a moderate-to-high level of infection). Herds with a BoHV-1 seroprevalence below 15% (deemed an indicator of a low level of infection) should be encouraged to incorporate strategic culling of seropositive animals over time, while maintaining vaccination and robust biosecurity practices to prevent external BoHV-1 exposure [30].

Vaccines used to control and eradicate BoHV-1 from the Irish cattle population must not only reduce clinical signs but also minimise viral shedding from latently infected animals to curb BoHV-1 transmission between infected and uninfected stock [31]. Hyperimmunisation, or vaccination every six months, effectively reduces the within-herd BoHV-1 seroprevalence over time [32]. While annual vaccination may control clinical disease, several studies have shown that hyperimmunisation protocols consistently reduce viral circulation and the number of seropositive animals over time [32–34]. On the other hand, evidence indicates that both annual vaccination and non-intervention are insufficient to reduce gE seroprevalence at the herd level [32, 35]. Large field studies assessing the impact of vaccination and biosecurity measures on the seroprevalence of BoHV-1 infection in RoI are limited and are needed to establish an effective approach in future national eradication plans [36].

This study aimed to assess the evolution of the BoHV-1 seroprevalence within a defined population of Irish dairy cattle at animal level, group level, and individual herd level, following the implementation of a hyperimmunisation IBR vaccination regimen. An annual biosecurity survey was also designed to identify potential risk factors for BoHV-1 infection. Ultimately, these approaches were being assessed to determine their suitability for reducing BoHV-1 seroprevalence, which could complement the roadmap for BoHV-1 eradication in RoI.

## Materials and methods

### Study design and eligibility criteria

**Fig. 1:**
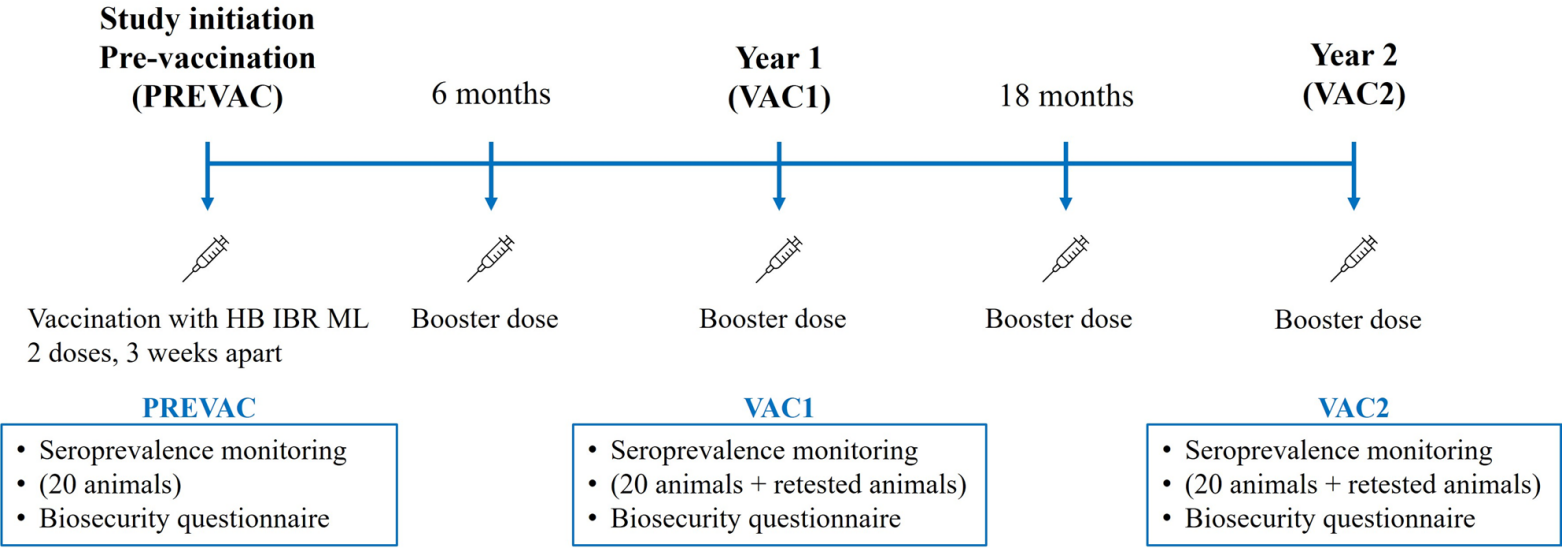
Diagram of the study design

An observational, prospective field study was conducted to evaluate the effect of vaccination on BoHV-1 seroprevalence in Irish dairy farms over a three-year period, from January 2020 to January 2023. Eligible farms participated by answering an annual biosecurity questionnaire and followed an IBR vaccination protocol against BoHV-1. Farms were offered continued serological surveillance and were monitored annually for two years. Information on culling and replacement rates was not available on the studied farms. A diagram with the study design is shown in Fig. 1.

Eligible farms were selected based on the following inclusion criteria: (1) Irish dairy herd with ≥ 80 animals, representing the average herd size in Ireland [27]; (2) a positive bulk milk tank IBR gE Enzyme Linked Immunosorbent Assay (ELISA) result, indicating BoHV-1 exposure; and (3) an estimated within-herd BoHV-1 seroprevalence of ≥ 25%. The enrolment process consisted of three phases:

#### Enrolment phase I: identification phase

Eligible dairy farms were identified by private veterinary practitioners (PVPs) from XLVets Ireland. These farms were investigated as part of routine surveillance, due to a lack of a BoHV-1 vaccination regimen, or due to concerns regarding the respiratory or reproductive health of the herd.

#### Enrolment phase II: investigation phase

The ‘*Investigation Phase*’ involved obtaining a bulk milk tank sample for submission through the XLVets network to a validated local laboratory accredited by the Irish National Accreditation Board (Farmlabs Diagnostic Ltd) for analysis (Bulk Milk Tank IBRgE ELISA, SOP548) [37, 38]. A positive ELISA result indicated moderate-to-high within-herd BoHV-1 seroprevalence, with at least 10–20% of the milking animals contributing to the tank being seropositive [38, 39]. Farms that tested positive proceeded to the next phase, while those with negative results were excluded. This phase provided commentary on the herd BoHV-1 infection status and an indirect estimate of the minimum within-herd BoHV-1 seroprevalence.

#### Enrolment phase III: recruitment phase

The ‘*Recruitment Phase*’ aimed to estimate the within-herd BoHV-1 seroprevalence and give an overview of the infection dynamics. A random sample of 20 female animals of varying age groups (from 6 months old to ≥ 24 months old) was tested. Herds with ≥ 25% estimated within-herd BoHV-1 seroprevalence were considered eligible to ensure a detectable effect during the study period. This decision was based on preliminary observations made during the ‘*Investigation Phase*’ of the enrolment process. Participating farms were required to implement a hyperimmunisation IBR vaccination protocol against BoHV-1 with a live, monovalent, double gene deleted (gE-/tk-) BoHV-1 strain CEDDEL vaccine (Laboratorios HIPRA S.A., Spain) [34, 40] and complete an annual biosecurity questionnaire.

### Biosecurity assessment

**Fig. 2:**
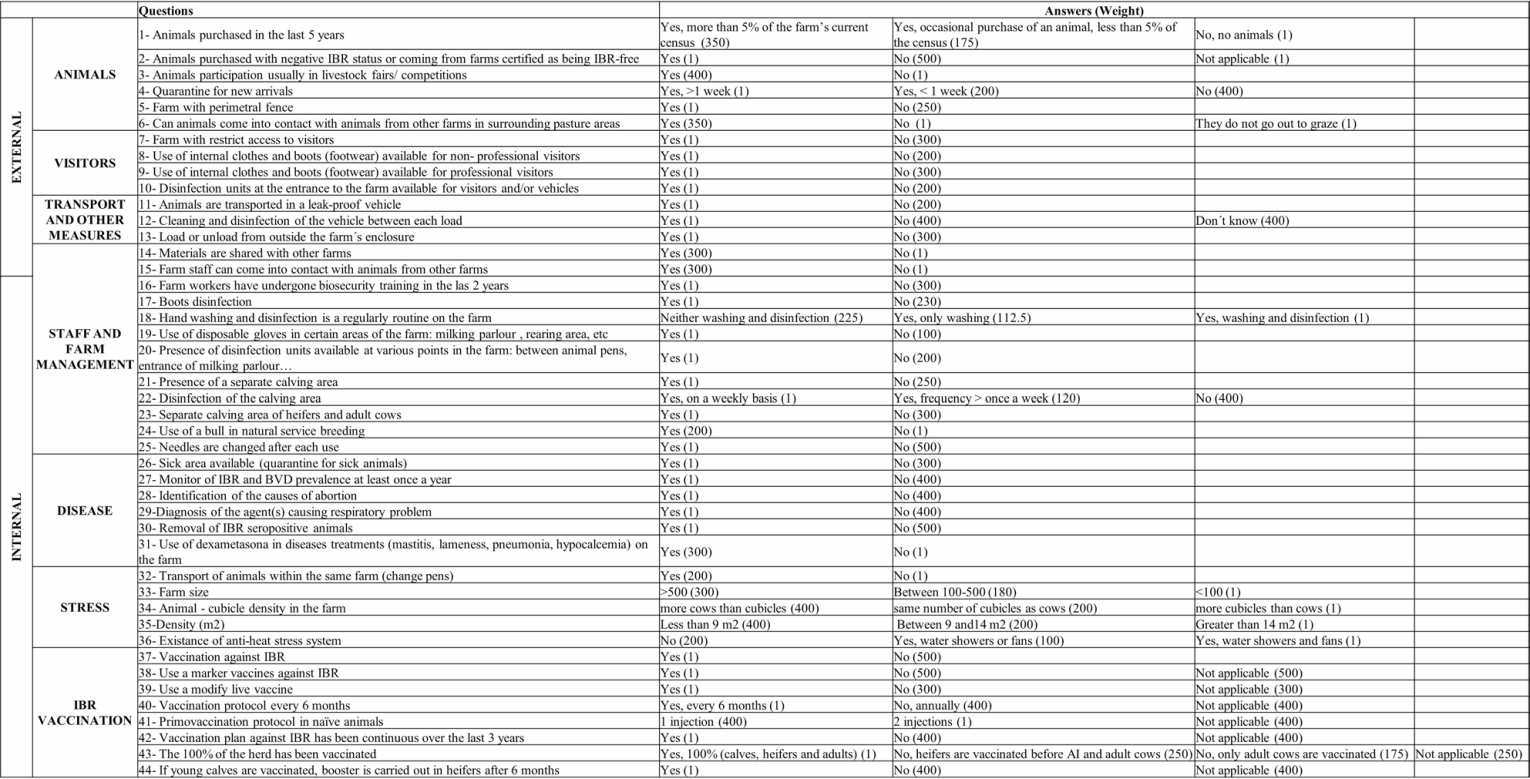
Biosecurity questionnaire

An annual biosecurity questionnaire was undertaken on each farm to identify potential risk factors for BoHV-1 infection and to determine areas where disease control measures could be improved over time. In this context, veterinarians provided biosecurity recommendations to farmers through an annual report, which outlined best practices based on the findings of the questionnaire. Each farm’s biosecurity was assessed at three timepoints: PREVAC, 12 months later (VAC1), and at 24 months (VAC2), using a 44-item questionnaire (Fig. 2). These questions focused on identifying biocontainment risk factors (31 questions) and bioexclusion risk factors (13 questions), classified into seven blocks. The blocks were assigned differing risk weightings towards an overall biosecurity score, based on the Delphi methodology used in a previous study [41].

Biocontainment (internal biosecurity) was queried in four of these blocks, namely ‘*Staff & Farm Management*’ (23% weighting), ‘*Disease*’ (16% weighting), ‘*Stress*’ (10% weighting), and ‘*IBR Vaccination*’ practices (22% weighting). Bioexclusion (external biosecurity) was queried in three blocks, namely ‘*Visitors*’ (7% weighting), ‘*Animals*’ (16% weighting), and ‘*Transport & Other Measures*’ (6% weighting) [41].

Internal, external, and overall biosecurity scores were calculated based on the responses to the 44-item questionnaire. Each answer was assigned a risk score (Fig. 2), and the internal, external, and overall biosecurity scores were computed as the sum of the risk scores for each relevant block, expressed as percentage relative to the maximum possible score using the following formula:

$$\text{Biosecurity score}\,\left(\%\right)=100\times\left(1-\frac{\sum \text{risk score}}{\text{Maximum score}}\right)$$

### Vaccination protocol

The recommended hyperimmunisation IBR vaccination regimen consisted of vaccination of the entire herd starting with calves from three months of age. The animals received an initial 2 mL intramuscular dose of the reconstituted vaccine. This was followed by a subsequent revaccination three weeks later with another 2 mL intramuscular injection, as per label. Following on from this primary vaccination course, a 2 mL intramuscular booster injection was administered every 6 months (Fig. 1).

### Serological assessment

BoHV-1 serological analyses were evaluated at the individual animal level during three timepoints, namely (i) PREVAC (ii) VAC1, and (iii) VAC2. All the individual blood samples were analysed using an IDEXX IBR gE enzymatic immunoassay (at HIPRA Diagnos, Amer, Girona, Spain), which differentiates animals exposed to wild-type BoHV-1 from those vaccinated with the marker vaccine [3]. This test allowed for accurate monitoring of natural exposure versus a vaccination response. Cut off values used in this laboratory were as follows: ≤0.6 represented a positive result (indicating previous wild BoHV-1 exposure), 0.6–0.7 represented an inconclusive result, and values > 0.7 indicated a negative result (indicating no previous wild BoHV-1 exposure).

### Herds and epidemiological units

**Fig. 3:**
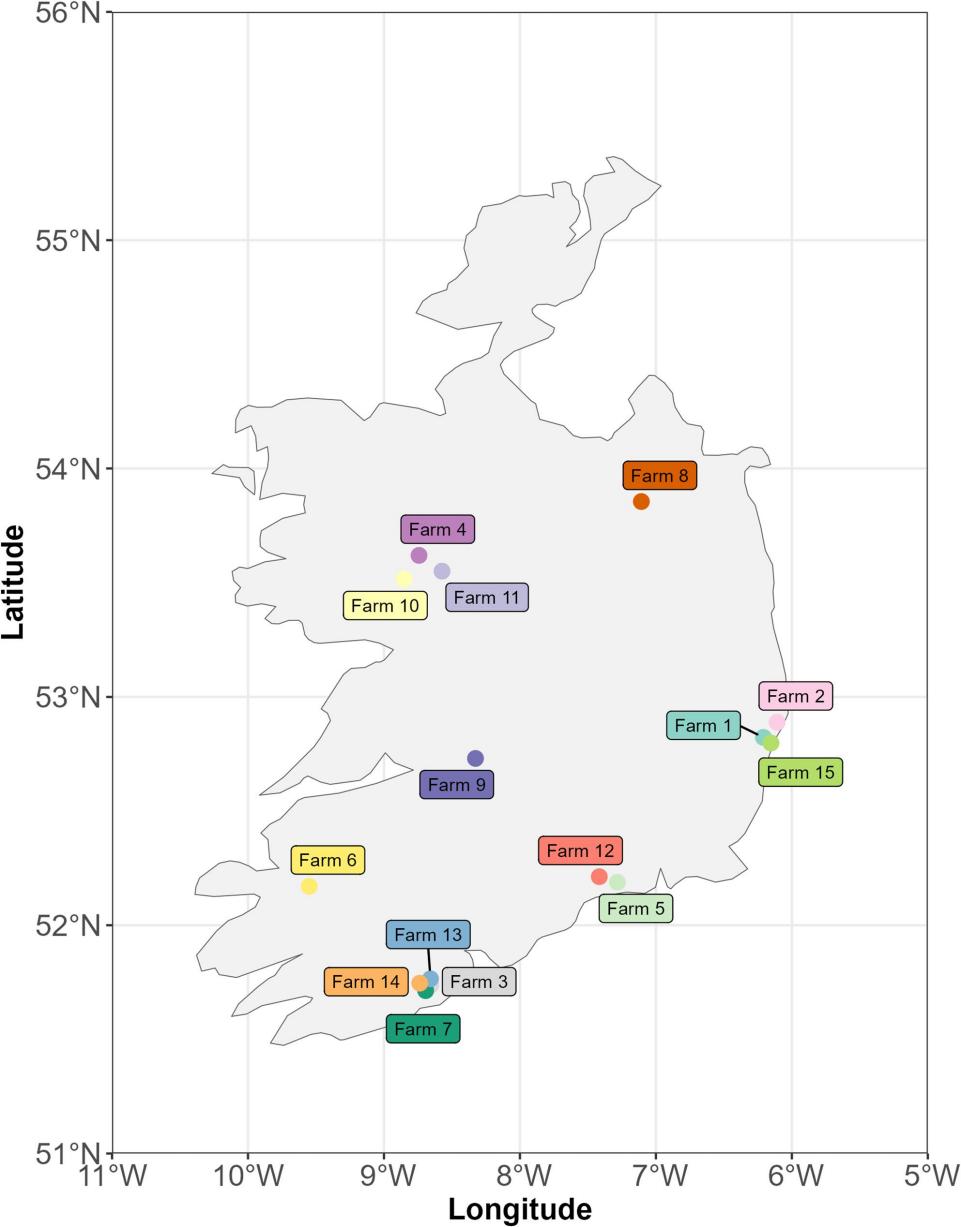
Distribution of the farms across the Republic of Ireland

Sixty-eight dairy farms were identified during the ‘*Identification Phase*’. Of the initial sixty-eight farms, thirty-five farms moved to the ‘*Recruitment Phase*’ based on their bulk milk tank results. Fifteen farms with herd sizes ranging from 85 to 250 animals over the study period (Table 1), met all further selection criteria and were invited to be involved. The distribution of the farms across RoI is shown in Fig. 3. Blood samples were collected annually from an average of 20 previously untested random animals per farm (range 16–28), divided into three epidemiological units based on age and sex: (i) ‘*Female Calves 6–12 Months Old*’ (CALF; 4 samples average, range 3–5), (ii) ‘*Replacement Heifers 12–24 Months Old*’ (HEIFER; 4 samples average, range 1–8), and (iii) ‘*Adult Dairy Cows ≥ 24 Months*’ (COW; 12 samples average, range 9–22). A total of 859 samples were assessed across the enrolled farms. Of note, young female calves were deemed eligible for sampling if they were destined to be retained within the future milking herd and aged between 6–12 months of age.

**Table 1:** Average size of the participating dairy herds

Farm	Animals
1	120
2	112
3	130
4	95
5	163
6	200
7	85
8	130
9	85
10	90
11	110
12	160
13	156
14	148
15	250
Total	2,034

Additionally, blood samples were also obtained and analyzed from animals that had returned a negative BoHV-1 serological result in the previous year(s). These animals were retested to investigate the vaccine’s ability to maintain a negative infection status, despite these animals residing in a seropositive herd with a moderate-to-high estimated within-herd BoHV-1 seroprevalence. A total of 175 samples from these animals were assessed across the enrolled farms. These animals were not included in the calculation of the estimated within-herd BoHV-1 seroprevalence.

### Statistical analysis

For the seroprevalence study, a logistic regression was performed where the explanatory variable was the BoHV-1 seroprevalence, and the response variables were year (PREVAC, VAC1 or VAC2) and the epidemiological unit (CALF, HEIFER or COW), and the farm was used as a random effect. Seroprevalence analysis in each farm was descriptive. To determine the correlation between estimated within-herd BoHV-1 seroprevalences and biocontainment scores, a Pearson correlation analysis was conducted.

For the biosecurity survey evaluation, a univariate logistic regression was performed for each individual question from the biosecurity questionnaire, where the response variable was the change in the BoHV-1 seroprevalence between years VAC1 and VAC2 (increase vs. decrease), and the explanatory variable was the risk level indicated by the answer to each questionnaire item (low, medium, or high). Statistical significance was set at a two-sided α < 0.05. Statistical analyses were performed using the R Studio software version 3.6.1 [42].

## Results

### Evolution: animal-level BoHV-1 seroprevalence

The animal level BoHV-1 seroprevalence within this population of animals was 55.7% at the PREVAC timepoint and decreased to 37.2% at VAC1, representing a significant relative reduction of 33.2% (*p* < 0.0001). By VAC2, the animal level BoHV-1 seroprevalence was maintained at 37.5% (Table 2).

**Table 2:** Serological results from the subjects over time on all farms (*n* = 15) and those that maintained a hyperimmunisation IBR vaccination protocol (*n* = 11)

	Positive animals/total (%; CI 95%)	Inconclusive animals/total (%; CI 95%)	Negative animals/total (%; CI 95%)	
All farms				*P* value
PREVAC	167/300 (55.7%; 49.8–61.3%)	2/300 (0.7%; 0.1–2.7%)	131/300 (43.7%; 38-49.5%)	Ref.
VAC1	111/298 (37.2%; 31.8–43%)	1/298 (0.3%; 0.02–2.2%)	186/298 (62.4%; 56.6–67.9%)	< 0.0001
VAC2	98/261 (37.5%; 31.7–43.8%)	1/261 (0.4%; 0.02–2.5%)	162/261 (62.1%; 55.9–67.9%)	< 0.0001
Farms that maintained the hyperimmunisation IBR protocol				*P* value
PREVAC	126/220 (57.3%;50.4–63.8%)	2/220 (0.9%; 0.2–3.6%)	92/220 (41.8%; 35.3–48.7%)	Ref.
VAC1	89/218 (40.8%; 34.3–47.7%)	1/218 (0.5%; 0.02–2.9%)	128/218 (58.7%; 51.9–65.3%)	0.0004
VAC2	77/229 (33.6%; 27.6–40.2%)	1/229 (0.4%; 0.02–2.8%)	151/229 (65.9%, 59.4–72%)	< 0.0001

*PREVAC* Pre-Vaccination, *VAC1* Vaccination Year One, *VAC2* Vaccination Year Two, *Ref* reference

Importantly, four herds no longer met the criteria for retention during the study period (either stopped vaccination or did not follow the hyperimmunisation protocol). Among the eleven dairy farms that maintained the hyperimmunisation IBR vaccination regimen, the animal level BoHV-1 seroprevalence decreased from 57.3% at PREVAC to 40.8% at VAC1 and to 33.6% at VAC2, representing an overall significant relative reduction of 41.4% (*p* < 0.0001) (Table 2). However, the reduction from VAC1 to VAC2 was minor, with overlapping 95% CIs indicating no significant change.

### Evolution: estimated within-herd BoHV-1 seroprevalence

The estimated within-herd BoHV-1 seroprevalence varied across farms. Although the overlapping 95% CIs indicate a lack of statistical significance, most of the farms (12 of 15) experienced a reduction in estimated within-herd BoHV-1 seroprevalence from PREVAC to VAC1 (mean relative reduction of 41.6%, from 57.9% to 33.8%). In half of the farms, this decrease was maintained (Farm 12) or further reduced by VAC2 (Farms 1, 3, 8, 14 and 15), all of which maintained the recommended hyperimmunisation IBR vaccination protocol. However, despite the overall decrease in BoHV-1 seroprevalence from PREVAC to VAC2, three of these twelve farms (5, 11 and 13), which also maintained the hyperimmunisation IBR vaccination protocol, showed an increase in the estimated seroprevalence from VAC1 to VAC2.

Importantly, four farms reached an estimated within-herd BoHV-1 seroprevalence < 25% by VAC2 (Farms 1, 3, 4, and 5). Notably, Farm 10 achieved an estimated within-herd BoHV-1 seroprevalence < 15% by VAC1 but this farm did not participate further, and Farm 6 had an estimated within-herd BoHV-1 seroprevalence of 20% by VAC1 but subsequently diverged from the recommended hyperimmunisation IBR vaccination protocol, and the seroprevalence increased to 75% by VAC2 (Table 3).

**Table 3:** Changes in apparent within-herd BoHV-1 seroprevalence between PREVAC and VAC2 of all participating farms

Farm	PREVAC (%; CI 95%)	VAC1 (%; CI 95%)	Percentage Difference with PREVAC	VAC2 (%; CI 95%)	Percentage Difference with VAC1
1	55.0% (32-76.2%)	30% (12.8–54.3%)	-25%	20% (6.6–44.3%)	-10%
2	80.0% (55.7–93.4%)	42.9% (22.6–65.6%)	-37.1%		
3	45.0% (23.8–68%)	36.8% (17.2–61.4%)	-8.2%	23.8% (9.1–47.5%)	-13%
4	55.0% (32-76.2%)	64.7% (38.6–84.7%)	+ 9.7%	21.1% (7-46.1%)	-43.7%
5	25.0% (9.6–49.4%)	5% (0.3–26.9%)	-20%	9.5% (1.7–31.8%)	+ 4.5%
6	65.0% (40.9–83.7%)	20% (6.6–44.3%)	-45%	75% (47.4–91.7%)	+ 55%
7	35.0% (16.3–59.1%)	40% (20-63.6%)	+ 5%	56.3% (30.6–79.2%)	+ 16.3%
8	80.0% (55.7–93.4%)	47.4% (25.2–70.5%)	-32.6%	30% (12.8–54.3%)	-17.4%
9	50.0% (29.9–70.1%)	50% (29.9–70.1%)	0%	68.4% (43.5–86.4%)	+ 18.4%
10	25.0% (9.6–49.4%)	5.3% (0.3–28.1%)	-19.7%		
11	50.0% (29.9–70.1%)	25% (9.6–49.4%)	-25%	28.6% (14-48.9%)	+ 3.6%
12	70.0% (45.7–87.2%)	47.6% (26.4–69.7%)	-22.4%	47.6% (26.4–69.7%)	0%
13	45.0% (23.8–68%)	38.1% (19-61.3%)	-6.9%	50% (29.9–70.1%)	+ 11.9%
14	70.0% (45.7–87.2%)	55% (32-76.2%)	-15%	40% (20-63.6%)	-15%
15	85.0% (61.1–96%)	52.4% (30.3–73.6%)	-32.6%	35% (16.3–59.1%)	-17.4%

*PREVAC* Pre-Vaccination, *VAC1* Vaccination Year One, *VAC2* Vaccination Year Two. Percentage difference between PREVAC and VAC1, and percentage difference between VAC1 and VAC2 (negative indicating reduction and positive indicating increase)

### Evolution: epidemiological unit BoHV-1 seroprevalence

At PREVAC, the results showed that BoHV-1 seropositivity increased with age, with cows showing the highest seropositivity compared to calves (*p* < 0.0001, Table 4). Over the two-years period, the reduction in BoHV-1 seropositivity in the fifteen participating farms varied according to the defined epidemiological units, with an overall 66.7% relative reduction in the CALF group (from 19.2% to 6.4%), a 70.6% relative reduction in the HEIFER group (from 25.5% to 7.5%), and a 23.8% relative reduction in the COW group (from 74.1% to 56.5%).

**Table 4:** Serological results from all animals throughout the study in the three epidemiological units on all farms (*n* = 15) and those that maintained a hyperimmunisation IBR vaccination protocol (*n* = 11)

	CALF			HEIFER			COW			
	Positive animals/total (%; CI 95%)	Inconclusive animals/total (%; CI 95%)	Negative animals/total (%; CI 95%)	Positive animals/total (%; CI 95%)	Inconclusive animals/total (%; CI 95%)	Negative animals/total (%; CI 95%)	Positive animals/total (%; CI 95%)	Inconclusive animals/total (%; CI 95%)	Negative animals/total (%; CI 95%)	
All farms										*P* value
PREVAC	10/52 (19.2%; 10.1–33%)	1/52 (1.9%; 0.1–11.6%)	41/52 (78.8%; 64.9–88.4%)	14/55 (25.5%; 15.1–39.3%)	1/55 (1.8%; 0.09-11%)	40/55 (72.7%; 58.8–83.5%)	143/193 (74.1%; 67.2–80%)	0/193 (0%)	50/193 (25.9%; 20-32.8%)	Ref.
VAC1	1/49 (2%; 0.1–12.2%)	1/49 (2%; 0.1–12.2%)	47/49 (96%; 84.9–99.3%)	2/66 (3%; 0.5–11.5%)	0/66 (0%)	64/66 (97%; 85.5–99.5%)	108/183 (59%; 51.5–66.1%)	0/183 (0%)	75/183 (41%; 33.9–48.5%)	< 0.0001
VAC2	3/47 (6.4%; 1.7–18.6%)	0/47 (0%)	44/47 (93.6%; 81.4–98.3%)	4/53 (7.5%; 2.4–19.1%)	1/53 (1.9%; 0.1–11.4%%)	48/53 (90.6%; 78.6–96.5%)	91/161 (56.5%; 48.5–64.2%)	0/161 (0%)	70/161 (43.5%; 35.8–51.5%)	< 0.0001
* P* value	Ref.			0.501			< 0.0001			
Farms that maintained a hyperimmunisation IBR vaccination protocol										*P* value
PREVAC	10/40 (25%; 13.2–41.5%)	1/40 (2.5%; 0.1–14.7%)	29/40 (72.5%; 55.9–84.9%)	7/38 (18.4%; 8.3–34.9%)	1/38 (2.6%; 0.1–15.4%)	30/38 (78.9%; 62.2–89.9%)	109/142 (76.8%; 68.8–83.3%)	0/142 (0%)	33/142 (23.2%; 16.7–31.2%)	Ref.
VAC1	1/37 (2.7%; 0.1–15.8%)	1/37 (2.7%; 0.1–15.8%)	35/37 (94.6%; 80.5–99.1%)	2/49 (4.1%; 0.7–15.1%)	0/49 (0%)	47/49 (95.9%; 84.9–99.3)	86/132 (65.2%; 56.3–73.1%)	0/132 (0%)	46/132 (34.8%; 26.9–43.7%)	< 0.0001
VAC2	3/43 (7.0%; 1.8–20.1%)	0/43 (0%)	40/43 (93.0%; 79.9–98.2%)	2/46 (4.3%; 0.8–16%)	1/46 (2.2%; 0.1–13%)	43/46 (93.5%; 81.1–98.3%)	72/140 (51.4%; 42.9–59.9%)	0/140 (0%)	68/140 (48.6%; 40.1–57.1%)	< 0.0001
* P* value	Ref.			0.364			< 0.0001			

*PREVAC* Pre-Vaccination, *VAC1* vaccination year One, *VAC2* vaccination year Two, *Ref* reference

Similar patterns were observed when only the herds which maintained the hyperimmunisation IBR vaccination protocol were considered, with marked decreases from PREVAC to VAC1 across all epidemiological units, but only minor reductions at VAC2 (Table 4).

### Evolution: maintenance of negative BoHV-1 infection status

To determine if individual animals with previous negative serological results maintained this negative-infection status, a total of 175 serological samples were obtained (69 at VAC1 and 106 at VAC2, Table 5). Overall, 151 samples remained seronegative (86.3%), with 91.3% at VAC1 and 83.0% at VAC2 (Table 6).

**Table 5:** Serological negative results in the animals re-assessed for BoHV-1 infection status at VAC1 and VAC2, presented as a percentage of total negative cases per participating farm

Farm	VAC1 Negative animals/total (%; CI 95%)	VAC2 Negative animals/total (%; CI 95%)
1*	4/5 (80%; 29.9–98.9%)	10/12 (83.3%; 50.9–97.1%)
2	-	-
3*	1/1 (100%; 5.5–100%)	12/12 (100%, 69.9–100%)
4	3/3 (100%; 31–100%)	3/3 (100%; 31–100%)
5*	11/12 (91.7%; 59.8–99.6%)	5/6 (83.3%; 36.5–99.1%)
6	3/3 (100%; 31–100%)	0/2 (0%; 0-80.2%)
7	9/10 (90%; 54.1–99.5%)	5/14 (35.7%; 14-64.4%)
8*	1/1 (100%; 5.5–100%)	2/2 (100%; 19.8–100%)
9*	5/5 (100%; 46.3–100%)	14/15 (93.3%; 66–100%)
10*	8/8 (100%; 59.8–100%)	-
11*	5/6 (83.3%; 36.5–99.1%)	4/4 (100%; 39.6–100%)
12*	4/4 (100%; 39.6–100%)	7/8 (87.5%; 46.7–99.3%)
13*	5/6 (83.3%; 36.5–99.1%)	7/7 (100%; 56.1–100%)
14*	4/4 (100%; 39.6–100%)	12/12 (100%, 69.9–100%)
15*	0% (0-94.5%) (0/1)	7/9 (77.7%; 40.2–96.1%)
Total samples re-assessed, n	69	106

*VAC1* Vaccination Year One, *VAC2* Vaccination Year Two

*Farms that maintained the hyperimmunisation IBR vaccination protocol throughout the study

**Table 6:** Overall serological results in animals reassesed for BoHV-1 infection status at VAC1 and VAC2 on all farms (*n* = 15) and on those that maintained the hyperimmunisation IBR protocol (*n* = 11) throughout the study

	VAC1	VAC2	Overall
All farms			
Seronegative/total (%; CI 95%)	63/69 (91.3%; 81.4–96.4%)	88/106 (83.0%; 74.2–89.4%)	151/175 (86.3%; 80.1–90.8%)
Inconclusive/total (%; CI 95%)	2/69 (2.9%; 0.5–11%)	2/106 (1.9%; 0.3–7.3%)	4/175 (2.3%; 0.7–6.1%)
Seropositive/total (%; CI 95%)	4/69 (5.8%; 1.9–14.9%)	16/106 (15.1%; 9.1–23.7%)	20/175 (11.4%;7.3–17.3%)
Farms that maintained the hyperimmunisation IBR protocol			
Seronegative/total (%; CI 95%)	43/48 (89.6%; 76.6–96.1%)	83/90 (92.2%; 84.1–96.5%)	126/138 (91.3%; 85-95.2%)
Inconclusive /total (%; CI 95%)	2/48 (4.2%; 0.7–15.4%)	2/90 (2.2%; 0.4–8.6%)	4/138 (2.9%; 0.9–7.7%)
Seropositive/total (%; CI 95%)	3/48 (6.25%; 1.6–18.2%)	5/90 (5.6%; 2.1–13.1%)	8/138 (5.8%; 2.7–11.5%)

*VAC1* Vaccination Year One, *VAC2* Vaccination Year Two

The eleven herds that maintained the recommended hyperimmunisation IBR vaccination protocol throughout the study showed similar patterns, with 91.3% of samples maintained seronegativity across the study (Table 6). Farm-level differences were evident: while some herds maintained a complete negative status among the samples collected (Farms 3, 4, 8 and 14), others showed higher seroconversion rates when the protocol was not consistently applied, particularly Farms 6 and 7 (Table 5).

During this study, it was noted that 2.4% (1/42) of the subjects classified within the CALF category which were reassessed as HEIFER had seroconverted. This contrasts with a figure of 13.1% (8/61) for subjects classified within the HEIFER category which were reassessed as COW. Interestingly, 87.5% (7/8) of these seroconversions occurred on farms that did not maintain the recommended hyperimmunisation IBR vaccination protocol (Farms 6 and 7). Furthermore, it was found that 13.9% (10/72) of subjects re-evaluated within the COW category seroconverted, with 50% (5/10) of these also occurring on those farms which did not follow the hyperimmunisation protocol (Farm 6 and Farm 7) between VAC1 and VAC2.

### Evolution of on-farm biosecurity

**Fig. 4:**
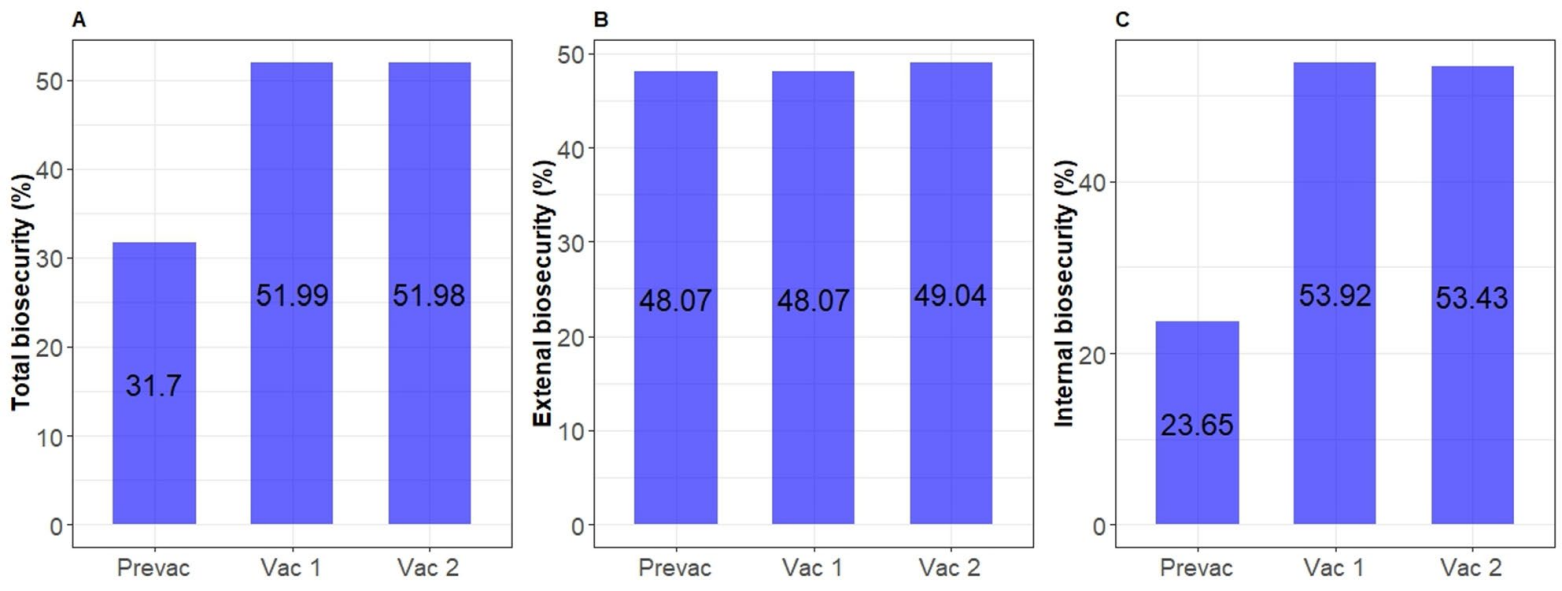
**A** Total biosecurity, **B** Bioexlusion, and **C** Biocontainment scores at PREVAC, Pre-Vaccination; VAC1, Vaccination Year One; and VAC2, Vaccination Year Two

Overall, biosecurity scores calculated based on the responses to the 44-item questionnaire increased from PREVAC to VAC1 but remained the same at VAC2 (Fig. 4A). The only consistent change in biocontainment measures was the implementation of an IBR vaccination regimen, which explained the overall increased biosecurity (Fig. 4C).

**Fig. 5:**
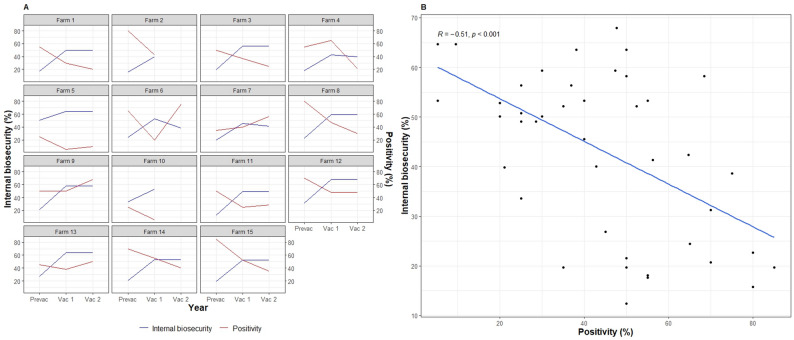
**A** Relationship between biocontainment scores and seropositivity at PREVAC, Pre-Vaccination; VAC1, Vaccination Year One; and VAC2, Vaccination Year Two. (*n* = 15). **B** Correlation between within-herd BoHV-1 seroprevalences and biocontainment scores

**Fig. 6:**
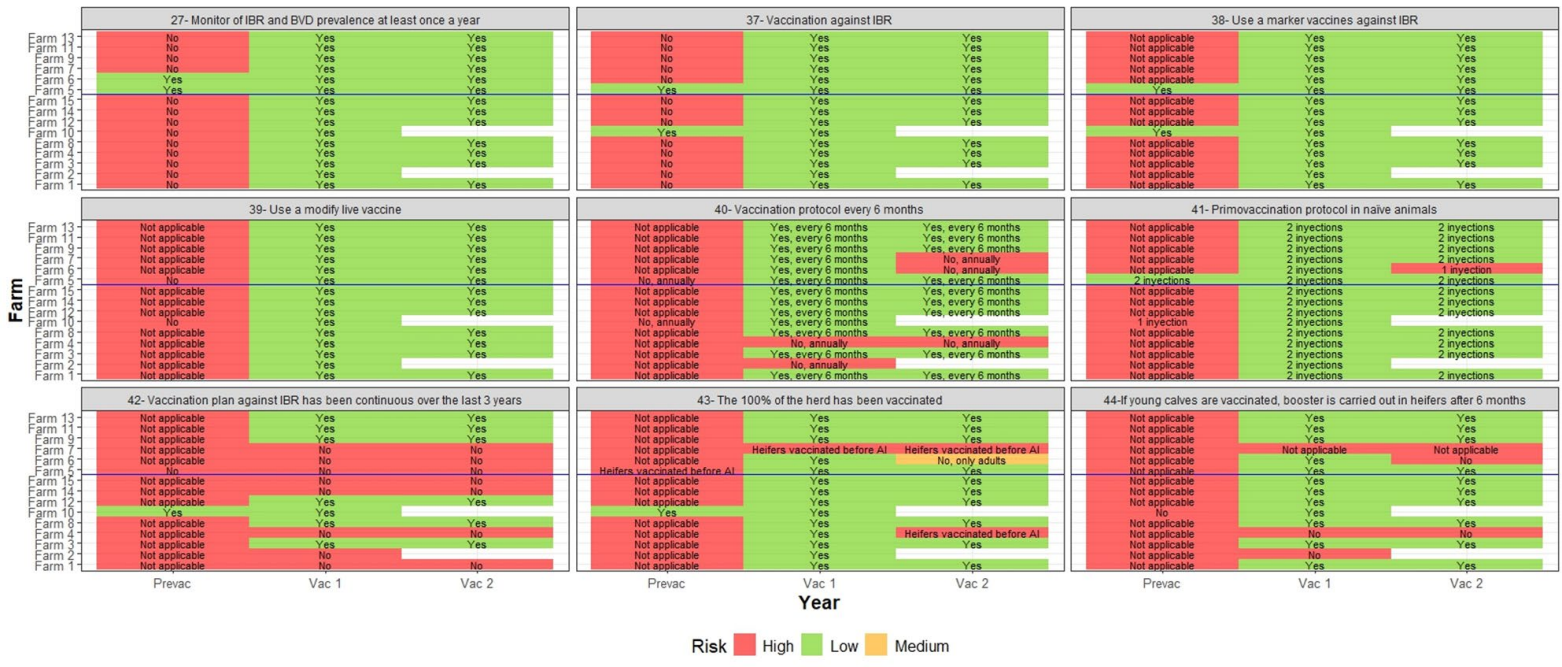
Biosecurity and answers to items 27 (monitoring) and 37 to 44 (IBR vaccination) in each participating farm. The farms above the blue line correspond to those with increased IBR seroprevalence the second year of vaccination respect first year

In general, biocontainment scores and estimated within-herd BoHV-1 seroprevalence showed opposite patterns between PREVAC and VAC1 in most of the farms (Fig. 5A), and were significantly and negatively correlated (*R* = -0.51, *p* < 0.001; Fig. 5B). Between VAC1 and VAC2, the biocontainment measures were stable, except for two dairy farms (Farms 6 and 7), where changes to ‘*IBR vaccination*‘ practices led to reduced biocontainment scores and increased BoHV-1 seroprevalence (Figs. 5A and 6). However, none of the questions related to the ‘*IBR Vaccination*‘ practices block had a significant association with an increase in seropositivity (see Additional file 1).

**Fig. 7:**
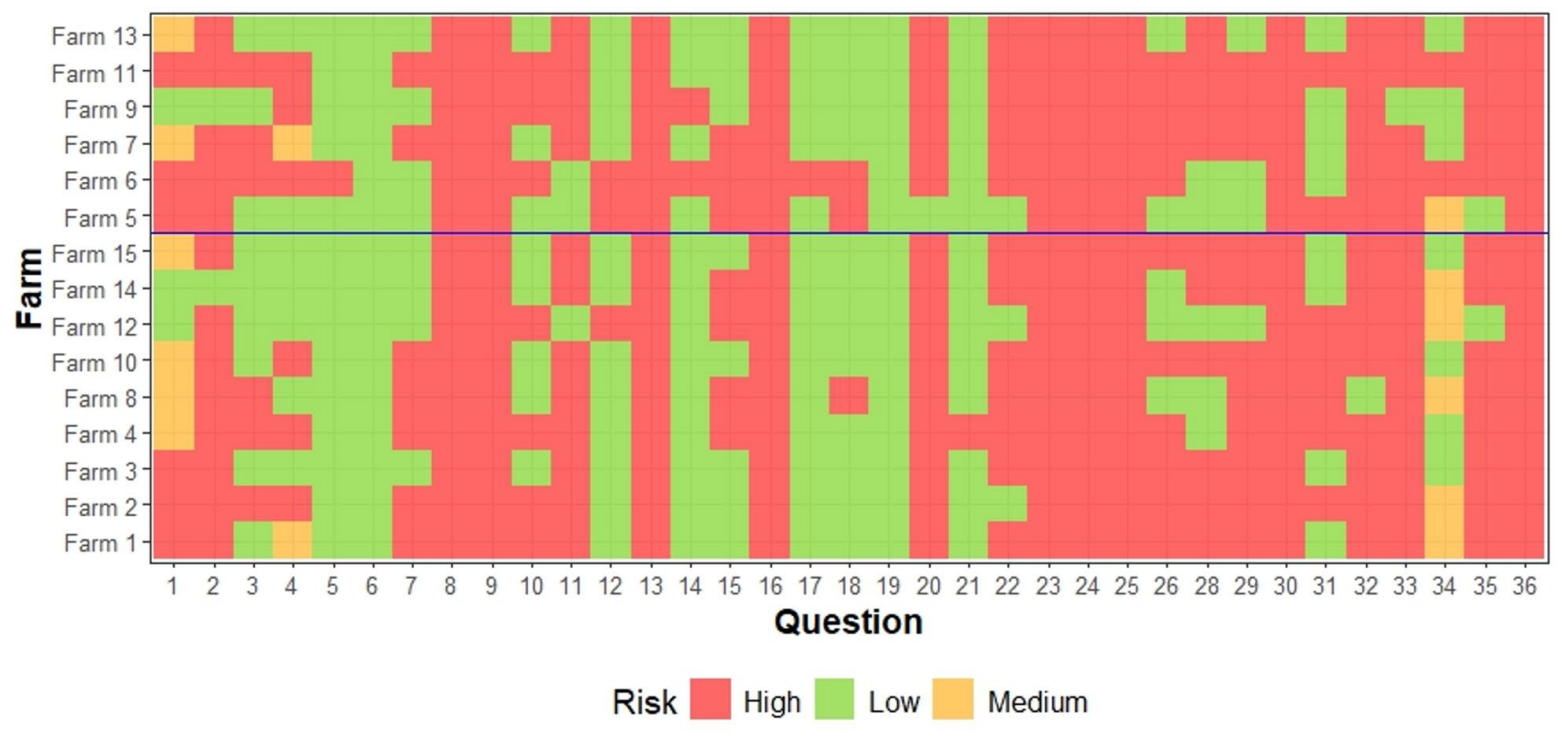
Biosecurity levels for the items included in the bioexclusion blocks: animals (Q1-6), visitors (Q7-10), and transport and other measures (Q11-13), and three of the biocontainment blocks: staff and farm management (Q14-25), disease (Q26-31), and stress (Q32-36). The farms above the blue line correspond to those with increased IBR seroprevalence the second year of vaccination respect first year

No major changes were observed in the remaining biocontainment blocks (‘*Staff & Farm Management*’, ‘*Disease*’, and ‘*Stress*’), nor in the bioexclusion blocks (‘*Visitors*’, ‘*Animals*’, and ‘*Transport & Other Measures*’). The responses and associated risk levels remained stable across all farms throughout the study period (Fig. 7). None of these questions reached significance in the regression analysis (see Additional file 1).

## Discussion

This study observed a notable reduction in BoHV-1 seroprevalence over a two-year period following the implementation of a hyperimmunisation IBR vaccination protocol (boosters every six months) using a live, double gene deleted marker (gE-/tk-) BoHV-1 strain CEDDEL vaccine. Across the eleven Irish dairy herds examined, animal-level BoHV-1 seroprevalence decreased from 57.3% to 33.6% (41.4% relative reduction), and the estimated average within-herd BoHV-1 seroprevalence from 54.5% to 22.2% (59.3% relative reduction). The most significant reduction occurred between PREVAC and VAC1 (35.6% on average, from 54.5% to 35.7%), and particularly among the CALF group aged 6–12 months (72.0%, from 25% to 7%) and the HEIFER group aged 12–24 months old (76.6%, from 18.4% to 4.3%). By the end of the study, one farm obtained a status of < 15%, the proposed cut-off for considering strategic culling as a control measure [30].

However, none of the biosecurity measures studied reached significance in the present study, possibly due to limitations in the sample size. Despite the wide 95% confidence intervals, some farms experienced larger declines in within-herd BoHV-1 seroprevalence, which may also have been influenced by the culling of seropositive animals (i.e., higher replacement rates) and/or the introduction of new seronegative calves. While these effects are plausible, the current study did not collect data on culling and replacement rates, limiting the ability to assess its exact impact. The findings should therefore be interpreted with caution, and further controlled studies with larger sample sizes, specifically in young animals, will be beneficial to confirm the impact of hyperimmunisation protocols under field conditions. Nonetheless, the results are consistent with previous studies in which hyperimmunization programmes led to a reduction in herd level seroprevalence [32–34].

While the present study observed a significant reduction in BoHV-1 seroprevalence during the first year, no further decline was detected the second year. Although a slight increase in seroprevalence was noticed in a few farms that followed the hyperimmunisation protocol, this variation was low and may be attributable to the limited sample size and the wide confidence intervals, which could have reduced the power to detect subtle epidemiological changes over time. However, the possibility of reintroduction of infection through replacement animals with unknown serological status, or ongoing viral circulation from latently infected carriers within the herd not detected in the re-assessed samples, cannot be excluded. Nevertheless, previous studies such as Rypula et al. [43]. reported a similarly rapid decline in within-herd BoHV-1 seroprevalence in one large herd from 65.7% to 39.0% in two years, and to < 4% in five years, using the same vaccine and protocol. This suggests that a longer vaccination and monitoring programme, joint with the management of infected animals (e.g. culling), may be necessary to further decrease BoHV-1 seroprevalence. Indeed, the long-term implementation of IBR vaccination protocols has led to BoHV-1 eradication in several herds, and DIVA IBR vaccination protocols have proven effective in eradicating the virus in many European regions. Therefore, DIVA IBR vaccination is a fundamental tool for eradication schemes [3, 44].

In addition to the overall trends observed, 91.3% of the animals re-assessed between PREVAC and VAC1 and VAC2, in the eleven dairy farms that maintained a hyperimmunisation IBR vaccination regimen, maintained a negative BoHV-1 infection status despite residing in a dairy herd with a previous moderate-to-high estimated within-herd BoHV-1 seroprevalence, indicative of a herd which had experienced widespread infection and uncontrolled viral circulation. A similar result was found in the 13 farms that followed the recommended vaccination protocol at VAC1. While these observations are encouraging, they must be interpreted with caution given the limited number of animals re-assessed. Although a significant negative correlation was observed between within-herd BoHV-1 seroprevalences and biocontainment scores, the logistic regression did not show a significant link between the hyperimmunisation IBR vaccination protocol and a decrease in seropositivity, likely due to the small sample size. Further investigation with a larger sample size would be beneficial to validate these assumptions.

Another finding was that, between VAC1 and VAC2, the two farms (Farm 6 and Farm 7) that did not follow the recommended hyperimmunisation IBR vaccination protocol experienced an increase in their estimated within-herd BoHV-1 seroprevalence, presenting a challenge to a future eradication programme. This aligns with the historical lack of progress in reducing dairy herd seroprevalence in RoI [11, 16] and corroborate previous observations [32, 35]. Both farms moved to an annual vaccination protocol between VAC1 and VAC2 and, additionally, Farm 7 did not complete the primary vaccination course between VAC1 and VAC2. Previous studies have shown that repeated vaccine administration (i.e., a 6-month inter-dose interval), is more effective, underscoring the importance of hyperimmunisation for efficacious IBR control [32], especially in farms with a moderate-to-high BoHV-1 within-herd seroprevalence. Moreover, neither farm vaccinated the entire herd between VAC1 and VAC2: Farm 6 only initiated a vaccination regime in replacement heifers and Farm 7 only vaccinated the adult dairy cows. Accordingly, the results indicate that failure to vaccinate 100% of the eligible animals within the herd is linked to an increase in estimated within-herd BoHV-1 seroprevalence. In this regard, previous studies have shown that two primary vaccinations in 3-month-old calves are critical for BoHV-1 control, particularly in young calves and naïve herds [32–34], suggesting that this approach, combined with frequent boosters, likely contributed to a decreasing BoHV-1 seroprevalence, especially in regions with a documented high herd BoHV-1 seroprevalence such as RoI [16]. However, increases were also observed in four farms that adhered to the hyperimmunisation protocol, denoting that herd-level factors beyond vaccination alone likely contributed to the variability observed between farms [32]. Ultimately, these findings highlight that, although adherence to a hyperimmunisation IBR vaccination protocol remains a key tool to control BoHV-1 circulation within herds, additional factors must also be considered.

Multiple studies have consistently reported highBoHV-1 seroprevalence in Irish dairy and beef herds, ranging from 75% to 80% [10–14, 45]. However, despite increasing vaccine sales [39], BoHV-1 seroprevalence has remained stable in a recent analysis of over > 17,000 Irish dairy herds, with no notable reduction in the proportion of herds with positive bulk tank results [16]. Although these figures are alarming and suggestive that current IBR mitigation measures may only be partially effective in reducing BoHV-1 seroprevalence, they primarily reflect herd BoHV-1 seroprevalence, where a herd is classified as positive if there is any evidence of infection, regardless of its extent. Over the past five years, there has been a consistent increase in IBR vaccination uptake [39], suggestive of an historically low base. For example, Cowley et al. [13] found that only 11–30% of Northern Irish herds had an IBR vaccination regime, while in 2009, only 1.8% of 1,113 herds in RoI had a vaccination regimen in place; by 2022, over 3.3 million doses of IBR vaccines were sold in RoI [16], compared to 470,000 doses in 2009 [12]. Since many of these IBR vaccines are licensed to reduce viral shedding, this control measure would be expected to contribute to a reduction in the proportion of latently infected animals within herds with evidence of exposure to BoHV-1 [16] and therefore, contribute to a reduction in herd BoHV-1 seroprevalence. However, progress on reducing the herd BoHV-1 seroprevalence in the Irish dairy sector has not been demonstrated to date.

Most national IBR eradication programmes typically rely on gE- marker IBR vaccines for reliable surveillance [3, 4]. The live, double gE-/tk-deleted BoHV-1 strain used in the current study has previously been associated with reduccions in within-herd seroprevalence under field conditions [46, 47]. Furthermore, live attenuated IBR vaccines have also been shown to induce superior protection compared to inactivated vaccines [48]. Therefore, these reported benefits should be considered when implementing a vaccination programme for BoHV-1 eradication. In the present study, the effectiveness of the IBR vaccination protocol has been critical in minimizing BoHV-1 circulation within herds. However, the overall reduction in BoHV-1 seroprevalence was less consistent between VAC1 and VAC2, with around half of the participating farms showing stable or further decreases, likely due to factors such as herd dynamics, variations of the immunization protocols, and differences in culling and replacement policies. For example, at PREVAC, the BoHV-1 seroprevalence was notably higher in the COW group (74.1%) compared to the HEIFER (25.5%) and CALF (19.2%) groups, highlighting the positive correlations between increasing age and herpesvirus infection [22, 49]. This was reflected in the seroconversion rates observed, with 13.9% (10/72) of COWs seroconverting after reassessment at VAC1 and VAC2, and 13.1% (8/61) of HEIFERs seroconverting after joining the adult milking herd, compared to just 2.4% (1/42) of CALFs seroconverting when reassessed as replacement heifers. Furthermore, in the majority of the farms that did not maintain the recommended hyperimmunisation IBR vaccination, the rates of seroconversion were substantially higher compared to those that followed the protocol. This dynamic likely explains the smaller reduction in seroprevalence observed in the VAC2, further emphasizing the importance of hyperimmunisation vaccination protocols in keeping animals free from infection. However, the speed at which seroprevalence decreases may largely depend on replacement policies (e.g., percentage of replacements per year, whether seropositive animals are selected for culling, etc.) [36]. While vaccination remains critical component of BoHV-1 control, these results highlight the need for a comprehensive approach that also includes improved biosecurity measures and strategic replacement practices.

In this sense, the annual biosecurity assessments revealed minimal changes on the participating farms throughout the study, with the only consistent change in biocontainment being the implementation of an IBR vaccination programme. None of the fifteen farms modified their bioexclusion policies, despite the known risks posed by the introduction of infected purchased cattle [50, 51]. This is a challenge, with 6.7 million cattle moved in 2016 in the RoI, making these herds vulnerable to pathogen transmission and disease spread [50, 52–54]. Interestingly, previous simulations [55] found that transport restrictions are effective in reducing herd BoHV-1 seroprevalence, demonstrating the benefits of maintaining a closed herd, or instigating a clear and quarantine policy.

Despite the lack of changes in biosecurity measures even with a substantial proportion of the bioexclusion items assessed in the questionnaire being at a high-risk level, the estimated within-herd BoHV-1 seroprevalence decreased on most farms, underscoring the potential contribution of the hyperimmunisation IBR vaccination programme. This suggests there is plenty of scope for improvements in the BoHV-1 biosecurity policies of Irish dairy herds [56]. Future studies should explore the barriers to improving biosecurity and further investigate factors influencing BoHV-1 seroprevalence changes after implementing a robust vaccination programme.

## Conclusions

The implementation and maintenance of a hyperimmunisation IBR vaccination protocol was associated with a notable reduction in the BoHV-1 seroprevalence in a defined cattle population and the estimated within-herd BoHV-1 seroprevalence in most of the Irish dairy herds studied, the first fundamental step in a national IBR eradication programme. Adherence to the hyperimmunisation IBR protocol was fundamental to achieve a decreased BoHV-1 seroprevalence. The results obtained herein suggest that hyperimmunisation protocols with a double-gene deleted (gE-/tk-) BoHV-1 strain CEDDEL vaccine help to successfully maintain a seronegative status under most circumstances. No other risk factors were found to be significantly associated with BoHV-1 seroprevalence, although other factors such as culling and replacement practices can play an important role. The findings of this study may have ramifications for the approach to national BoHV-1 eradication as the farms included represented an average Irish dairy farm regarding size, herd seropositivity, and within-herd BoHV-1 seroprevalence.

## Supplementary Information

Supplementary Material 1.

## Data Availability

The datasets generated and/or analysed during the current study are available from the corresponding author upon reasonable request.

## Abbreviations

AHI: Animal Health Ireland
BoHV-1: Bovine Herpesvirus-1
CSO: Central Statistics Office
DIVA: Differentiate Infected and Vaccinated Animals
ELISA: Enzyme Linked Immunosorbent Assay
EMA: European Medicines Agency
EU: European Union
gE: Glycoprotein E
IBR: Infectious Bovine Rhinotracheitis
IPB: Infectious Pustular Balanoposthitis
IPVV: Infectious Pustular Vulvovaginitis
MDA: Maternally Derived Antibodies
POM: Prescription Only Medicine
PREVAC: Pre-Vaccination
PVP: Private Veterinary Practitioner
R0: Basic Reproduction Number
RoI: Republic of Ireland
SOP: Standard Operating Procedure
tk: Thymidine Kinase
VAC1: Vaccination Year One
VAC2: Vaccination Year Two

## References

[1] Quinn PJ, Leonard FC, Markey BK, Fanning S, Fitzpatrick ES. Concise review of veterinary microbiology. 2nd ed. Wiley-Blackwell, John Wiley & Sons Ltd, West Sussex, UK; 2015.

[2] Graham DA. Bovine herpes virus-1 (BoHV-1) in cattle-a review with emphasis on reproductive impacts and the emergence of infection in Ireland and the united Kingdom. Ir Vet J BioMed Cent Ltd. 2013;66(1):15. 10.1186/2046-0481-66-15. 10.1186/2046-0481-66-15 PMC3750245 23916092

[3] Iscaro C, Cambiotti V, Petrini S, Feliziani F. Control programs for infectious bovine rhinotracheitis (IBR) in European countries: an overview. Anim Health Res Rev. 2021;22:136–46. 10.1017/S1466252321000116. 35076360 10.1017/S1466252321000116

[4] Ministerio de Agricultura, Pesca y Alimentación (MAPA). Real Decreto 554/2019, de 27 de septiembre, por el que se establecen las bases de las actuaciones de prevención, control y erradicación de la rinotraqueítis infecciosa bovina y se establece un programa nacional voluntario de lucha contra dicha enfermedad. 2019. https://www.boe.es/boe/dias/2019/10/11/pdfs/BOE-A-2019-14553.pdf . Accessed 21 Oct 2025.

[5] Animal Health Ireland (AHI). National IBR Eradication Programme. Business Plan. 2023. https://animalhealthireland.ie/assets/uploads/2023/02/AHI-BusPlans-2023-IBR-FINAL.pdf?dl=1. Accessed 19 Feb 2025.

[6] Animal Health Ireland (AHI). National IBR Eradication Programme. Business Plan. 2024. https://animalhealthireland.ie/assets/uploads/2024/02/AHI_BusPlans_2024_IBR_FINAL.pdf?dl=1. Accessed 19 Feb 2025.

[7] Animal Health Ireland (AHI). Strategic Plan 2022–2026. 2023. https://animalhealthireland.ie/assets/uploads/2023/02/AHI-SD_2022-2026_V1_Web_Version_FINAL.pdf. Accessed 19 Feb 2025.

[8] Guelbenzu M. National IBR Eradication Programme. Animal Health Ireland Annual Report 2022. 2022. https://animalhealthireland.ie/assets/uploads/2023/06/AHI_AR_2022_Final_Web_08_05_2022.pdf . Accessed 19 Feb 2025.

[9] Department of Agriculture, Food and the Marine. Biocidal Product Register. Available at: https://publicapps.agriculture.gov.ie/prs/home . Accessed 3 Oct. 2025.

[10] O’Grady L, O’Neill R, Collins DM, Clegg TA, More SJ. Herd and within-herd BoHV-1 prevalence among Irish beef herds submitting bulls for entry to a performance testing station. Ir Vet J. 2008;61:809–15. 10.1186/2046-0481-61-12-809. 21851705 10.1186/2046-0481-61-12-809 PMC3113875

[11] Sayers R. IBR: Does Ireland Need a Control Programme? Dairying: Entering a Decade of Opportunity Teagasc National Dairy Conference. 2010. p. 79–86.

[12] Cowley DB, Clegg TA, Doherty ML, More SJ. Aspects of bovine herpesvirus-1 infection in dairy and beef herds in the Republic of Ireland. Acta Vet Scand. 2011;53(1):40. 10.1186/1751-0147-53-40. 21699677 10.1186/1751-0147-53-40 PMC3141558

[13] Cowley DJB, Graham DA, Guelbenzu M, Doherty ML, More SJ. Aspects of bovine herpesvirus 1 and bovine viral diarrhoea virus herd-level Seroprevalence and vaccination in dairy and beef herds in Northern Ireland. Ir Vet J. 2014;67(1):18. 10.1186/2046-0481-67-18. 25152811 10.1186/2046-0481-67-18 PMC4141657

[14] Barrett D, Parr M, Fagan J, Johnson A, Tratalos J, Lively F, et al. Prevalence of bovine viral diarrhoea virus (BVDV), bovine herpes virus 1 (BHV 1), leptospirosis and Neosporosis, and associated risk factors in 161 Irish beef herds. BMC Vet Res. 2018;14(1):8. 10.1186/s12917-017-1324-9. 29304782 10.1186/s12917-017-1324-9 PMC5756399

[15] Animal Health Ireland (AHI). Stakeholders’ Seasonal Updates - Autumn 2022 Edition. 2022. https://animalhealthireland.ie/assets/uploads/2022/10/AHI-Stakeholders-Newsletter-Autumn-2022-FINAL.pdf?dl=1. Accessed 19 Feb 2025.

[16] Animal Health Ireland (AHI). Stakeholders’ Seasonal Updates - Autumn 2023 Edition. 2023. https://animalhealthireland.ie/assets/uploads/2023/10/AHI_Stakeholders_Q4_2023_V1_FINAL.pdf?dl=1. Accessed 19 Feb 2025.

[17] Snowdon WA. The IBR-IPV virus: reaction to infection and intermittent recovery of virus from experimentally infected cattle. Aust Vet J. 1965;41:135–42. 10.1111/j.1751-0813.1965.tb15386.x.

[18] Thiry E, Brochier B, Saliki J, Pirak M, Pastoret PP. Excretion and reexcretion of thermosensitive and wild-type strains of infectious bovine rhinotracheitis virus after co-infection or two successive infections. Vet Microbiol. 1985;10(4):371–80. 10.1016/0378-1135(85)90007-0. 2994280 10.1016/0378-1135(85)90007-0

[19] Thiry E, Saliki J, Bublot M, Pastoret PP. Reactivation of infectious bovine rhinotracheitis virus by transport. Comp Immunol Microbiol Infect Dis. 1987;10:59–63. 10.1016/0147-9571(87)90041-5. 3034502 10.1016/0147-9571(87)90041-5

[20] Animal Health Ireland (AHI). IBR Eradication Programme. IBR in Cattle. Frequently Asked Questions. IBR Eradication leaflet series. 2021. https://animalhealthireland.ie/assets/uploads/2021/07/IBR-FAQs-2021.pdf?dl=1. Accessed 19 Feb 2025.

[21] Muylkens B, Thiry J, Kirten P, Schynts F, Thiry E. Bovine herpesvirus 1 infection and infectious bovine rhinotracheitis. Vet Res. 2007;38(2):181–209. 10.1051/vetres:2006059. 17257569 10.1051/vetres:2006059

[22] Nettleton P, Russell G. Update on infectious bovine rhinotracheitis. Pract. 2017;39:255–72. 10.1136/inp.j2226.

[23] Hage JJ, Schukken YH, Barkema HW, Benedictus G, Rijsewijk FAM, Wentink GH. Population dynamics of bovine herpesvirus 1 infection in a dairy herd. Vet Microbiol. 1996;53(1–2):169–80. 10.1016/s0378-1135(96)01245-x. 9011009 10.1016/s0378-1135(96)01245-x

[24] Department of Agriculture F and the M. Food Harvest. 2020. https://www.gov.ie/en/publication/5a0f2-food-harvest-2020/. Accessed 19 Feb 2025.

[25] Livestock Survey December 2013 - CSO - Central Statistics Office. 2013. https://www.cso.ie/en/releasesandpublications/er/lsd/livestocksurveydecember2013/. Accessed 19 Feb 2025.

[26] Livestock Survey December 2023 - Central Statistics Office. 2023. https://www.cso.ie/en/releasesandpublications/ep/p-lsd/livestocksurveydecember2023/. Accessed 19 Feb 2025.

[27] Teagasc National Farm Survey 2022. 2022. https://www.teagasc.ie/media/website/publications/2023/NFSfinalreport2022.pdf . Accessed 19 Feb 2025.

[28] Woodbine KA, Medley GF, Moore SJ, Ramirez-Villaescusa AM, Mason S, Green LE. A four year longitudinal sero-epidemiological study of bovine herpesvirus type-1 (BHV-1) in adult cattle in 107 unvaccinated herds in South West England. BMC Vet Res. 2009;5:5. 10.1186/1746-6148-5-5. 19183476 10.1186/1746-6148-5-5 PMC2657118

[29] Barrett D, Lane E, Lozano JM, O’Keeffe K, Byrne AW. Bovine herpes virus type 1 (BoHV-1) seroprevalence, risk factor and bovine viral diarrhoea (BVD) co-infection analysis from Ireland. Sci Rep. 2024;14:869. 10.1038/s41598-023-50433-5. 38195809 10.1038/s41598-023-50433-5 PMC10776861

[30] Animal Health Ireland (AHI). Annual Report 2021. 2021. animalhealthireland.ie/assets/uploads/2022/05/AHI__AR_2021_V1_FINAL_Web_Version.pdf. Accessed 19 Feb 2025.

[31] Van Oirschot JT. Diva vaccines that reduce virus transmission. J Biotechnol. 1999;73(2–3):195–205. 10.1016/s0168-1656(99)00121-2. 10486928 10.1016/s0168-1656(99)00121-2

[32] Ampe B, Duchateau L, Speybroeck N, Berkvens D, Dupont A, Kerkhofs P, et al. Assessment of the long-term effect of vaccination on transmission of IBR virus in cattle herds hyperimmunized with glycoprotein E-deleted marker vaccine. Am J Vet Res. 2012;73(11):1787–93. 10.2460/ajvr.73.11.1787. 23106465 10.2460/ajvr.73.11.1787

[33] Bosch JC, De Jong MCM, Franken P, Frankena K, Hage JJ, Kaashoek MJ, et al. An inactivated gE-negative marker vaccine and an experimental gD-subunit vaccine reduce the incidence of bovine herpesvirus 1 infections in the field. Vaccine. 1998;16(2–3):265–71. 10.1016/s0264-410x(97)00166-7. 9607041 10.1016/s0264-410x(97)00166-7

[34] Mars MH, De Jong MCM, Franken P, Van Oirschot JT. Efficacy of a live glycoprotein E-negative bovine herpesvirus 1 vaccine in cattle in the field. Vaccine. 2001;19(15–16):1924–30. 10.1016/s0264-410x(00)00435-7. 11228362 10.1016/s0264-410x(00)00435-7

[35] Dispas M, Lemaire M, Speybroeck N, Berkvens D, Dupont A, Boelaert F, Dramaix M, Vanopdenbosch E, Kerkhofs P, Thiry E. Deux protocoles d’hyperimmunisation Au Moyen de vaccins marqués réduisent l’incidence de séroconversion Envers l’herpèsvirus Bovin 1 En cheptels laitiers: résultats d’une étude Sur Le terrain. Ann De Médecine Vétérinaire. 2004;148(1):47–61.

[36] Brock J, Lange M, Guelbenzu-Gonzalo M, Meunier N, Vaz AM, Tratalos JA, et al. Epidemiology of age-dependent prevalence of bovine herpes virus type 1 (BoHV-1) in dairy herds with and without vaccination. Vet Res. 2020;51(1):124. 10.1186/s13567-020-00842-5. 32988417 10.1186/s13567-020-00842-5 PMC7520977

[37] FarmLab Diagnostics Ltd. Schedule of Accreditation. http://www.farmlab.ie. Accessed 19 Feb 2025.

[38] Farmlab Diagnostics Ltd. Farmlab Sample Submission Form. https://www.farmlab.ie/farmlab-sample-submission/. Accessed 19 Feb 2025.

[39] Guelbenzu M. The role of bulk tank milk testing in monitoring BVD and IBR. Vet Ire J. 2022;12:325–9. https://veterinaryirelandjournal.com/images/2022/june2022/pdf/la_june2022.pdf. Accessed 19 Feb 2025.

[40] European Medicines Agency (EMA). Hiprabovis IBR Marker Live^®^. https://www.ema.europa.eu/en/medicines/veterinary/EPAR/hiprabovis-ibr-marker-live. Accessed 19 Feb 2025.

[41] Santo Tomas H, Guix R, Nodar L, Couper A. IBR biosecurity survey. British Cattle Veterinary Association (BCVA) Congress , Newport, UK; 2021.

[42] R Core Team. R: A language and environment for statistical computing. R foundation for Statistical Computing, Vienna, Austria. 2021. Available from: https://www.R-project.org/.

[43] Rypuła K, Ehr N, Karuga-Kuźniewska E, Ptak W. Effect of vaccination with a modified -live vaccine (HIPRABOVIS IBR MARKER LIVE) on eradication of BoHV-1 infection on a dairy farm - Economic impact on production and clinical problems. European Buiatrics Forum (EBF), Bilbao, Spain ; 2017.

[44] Makoschey B, Zehle HH, Bussacchini M, Valla G, Pálfi V, Földi J. Efficacy of a live bovine herpesvirus type 1 marker vaccine under field conditions in three countries. Vet Rec. 2007;161:295–8. 10.1136/vr.161.9.295. 17766807 10.1136/vr.161.9.295

[45] Sayers RG. Associations between exposure to bovine herpesvirus 1 (BoHV-1) and milk production, reproductive performance, and mortality in Irish dairy herds. J Dairy Sci. 2017;100(2):1340–52. 10.3168/jds.2016-11113. 27939532 10.3168/jds.2016-11113

[46] Santo Tomas H, Turón Quer L, Ordis P, Mato I, Barreto M. Efficacy of Blocking IBR Viral Circulation Using a Double-Deleted GE-/Tk- Vaccine on a Dairy Farm. 101st Conference of Research Workers on Animal Diseases (CRWAD). 2020;Virtual: https://crwad.org/wp-content/uploads/CRWAD-2020-Proceedings-FINAL.pdf Accessed 19 Feb 2025.

[47] Pereira do Rio A, Fernandes da Silva D. Efficacy in the Reduction of IBR Prevalence When a Marker Live GE-/Tk-Deleted Vaccine Was Used Following an Outbreak in a Dairy Farm in Northern Portugal. 101st Conference of Research Workers on Animal Diseases (CRWAD). 2020;Virtual: https://crwad.org/wp-content/uploads/CRWAD-2020-Proceedings-FINAL.pdf . Accessed 19 Feb 2025.

[48] Bosch J, Kaashoek M, Kroese A, van Oirschot J. An attenuated bovine herpesvirus 1 marker vaccine induces better protection than two inactivated marker vaccines. Vet Microbiol. 1996;52(3–4):223–34. 10.1016/s0378-1135(96)00070-3. 8972048 10.1016/s0378-1135(96)00070-3

[49] Toomer G, Workman A, Harrison KS, Stayton E, Hoyt PR, Jones C. Stress triggers expression of bovine herpesvirus 1 infected cell protein 4 (bICP4) RNA during early stages of reactivation from latency in pharyngeal tonsil. J Virol. 2022;96(23):e0101022. 10.1128/jvi.01010-22. 36416585 10.1128/jvi.01010-22 PMC9749472

[50] McGrath G, Tratalos JA, More SJ. A visual representation of cattle movement in Ireland during 2016. Ir Vet J. 2018;71:18. 10.1186/s13620-018-0129-x. 30202515 10.1186/s13620-018-0129-x PMC6128986

[51] Mee JF, Geraghty T, O’Neill R, More SJ. Bioexclusion of diseases from dairy and beef farms: risks of introducing infectious agents and risk reduction strategies. Vet J. 2012;194(2):143–50. 10.1016/j.tvjl.2012.07.001. 23103219 10.1016/j.tvjl.2012.07.001 PMC7110757

[52] Strain S, Verner S, Campbell E, Hodnik JJ, Santman-Berends IMGA. The Northern Ireland control programmes for infectious cattle diseases not regulated by the EU. Front Vet Sci. 2021;8:694197. 10.3389/fvets.2021.694197. 34513968 10.3389/fvets.2021.694197 PMC8427759

[53] O’donnell S, Tyrrell J. Farm Fragmentation in Irish Dairying Overcoming and Adapting to it. A report for NUFFIELD IRELAND Farming Scholarships. 2013. Online: https://www.nuffieldscholar.org/sites/default/files/reports/2013_IE_Sean-Odonnell_Farm-Fragmentation-In-Irish-Dairying-Overcoming-And-Adapting-To-It.pdf Accessed 19 Feb 2025.

[54] Ashe S, More SJ, O’Keeffe J, White P, McGrath G, Aznar I. Survival and dispersal of a defined cohort of Irish cattle. Ir Vet J. 2009;62(1):44–9. 10.1186/2046-0481-62-1-44. 21851724 10.1186/2046-0481-62-1-44 PMC3113782

[55] Brock J, Lange M, Tratalos JA, More SJ, Guelbenzu-Gonzalo M, Graham DA, et al. A large-scale epidemiological model of BoHV-1 spread in the Irish cattle population to support decision-making in conformity with the European animal health law. Prev Vet Med. 2021;192:105375. 10.1016/j.prevetmed.2021.105375. 33989913 10.1016/j.prevetmed.2021.105375

[56] Sayers RG, Sayers GP, Mee JF, Good M, Bermingham ML, Grant J, et al. Implementing biosecurity measures on dairy farms in Ireland. Vet J. 2013;197(2):259–67. 10.1016/j.tvjl.2012.11.017. 23276712 10.1016/j.tvjl.2012.11.017

