# Supplementary material for: Evolution of Bovine Herpesvirus-1 infection prevalence and infection dynamics in Irish dairy herds following an IBR hyperimmunisation vaccination protocol
Source: Ir Vet J. 2025 Dec 23;79:10. doi: 10.1186/s13620-025-00328-w (PMC12853841; doi:10.1186/s13620-025-00328-w)
Supplement: Supplementary file 1 — Supplementary Material 1. [file 13620_2025_328_MOESM1_ESM.docx]

**Additional file 1.** Logistic regression analysis of biosecurity questions (response variable: change in the BoHV-1 seroprevalence between VAC1 and VAC2; explanatory variable: risk level of each response).

| **Question** | **OR** | **Confidence interval** | ***P* value** | **Question** | **OR** | **Confidence interval** | ***P* value** |
| --- | --- | --- | --- | --- | --- | --- | --- |
| 1 | 0.33 | 0.01-6.15 | 0.472 | 23 | - | NC | - |
| 2 | 1.20 | 0.40-35.97 | 0.906 | 24 | - | NC | - |
| 3 | 0.40 | 0.03-3.85 | 0.433 | 25 | - | NC | - |
| 4 | 0.13 | 0.00-1.77 | 0.158 | 26 | 0.67 | 0.06-6.43 | 0.725 |
| 5 | - | NC | 0.996 | 27 | - | NC | - |
| 6 | - | NC | - | 28 | 0.67 | 0.06-6.43 | 0.725 |
| 7 | 1.50 | 0.16-16.80 | 0.725 | 29 | 6.00 | 0.51-155.19 | 0.186 |
| 8 | - | NC | - | 30 | - | NC | - |
| 9 | - | NC | - | 31 | 1.50 | 0.16-16.80 | 0.725 |
| 10 | 0.75 | 0.08-6.93 | 0.797 | 32 | - | NC | 0.996 |
| 11 | 3.00 | 0.21-78.76 | 0.427 | 33 | - | NC | 0.996 |
| 12 | 0.33 | 0.01-4.68 | 0.427 | 34 | - | NC | 0.997 |
| 13 | - | NC | - | 35 | 1.20 | 0.40-35.97 | 0.906 |
| 14 | - | NC | 0.997 | 36 | - | NC | - |
| 15 | 1.33 | 0.14-12.95 | 0.797 | 37 | - | NC | - |
| 16 | - | NC | - | 38 | - | NC | - |
| 17 | - | NC | 0.996 | 39 | - | NC | - |
| 18 | 0.33 | 0.01-4.68 | 0.427 | 40 | 0.33 | 0.01-4.68 | 0.427 |
| 19 | - | NC | - | 41 | - | NC | 0.996 |
| 20 | - | NC | 0.996 | 42 | 1.33 | 0.14-12.95 | 0.797 |
| 21 | - | NC | 0.996 | 43 | 0.67 | 0.02-20.33 | 0.794 |
| 22 | 1.20 | 0.04-35.97 | 0.906 | 44 | 0.33 | 0.01-4.68 | 0.427 |

OR, Odds ratio (Low risk vs High risk). NC, not computed.

.
